# 1674. Trends in Antimicrobial Susceptibility to Ceftolozane/Tazobactam and Comparators of *Pseudomonas aeruginosa* from Patients with Respiratory Tract Infections in Five Latin American Countries – SMART 2017-2020

**DOI:** 10.1093/ofid/ofac492.1304

**Published:** 2022-12-15

**Authors:** Sibylle Lob, Meredith Hackel, Fakhar Siddiqui, Jacqueline Pavia, Charles A DeRyke, Katherine Young, Mary Motyl, Daniel F Sahm

**Affiliations:** Merck & Co., Inc., Schaumburg, Illinois; IHMA, Schaumburg, Illinois; Merck & Co., Inc., Schaumburg, Illinois; MSD Colombia, Bogota, Colombia, Bogota, Distrito Capital de Bogota, Colombia; Merck Research Laboratories, Longwood, Florida; Merck, Rahway, New Jersey; Merck, Rahway, New Jersey; IHMA, Schaumburg, Illinois

## Abstract

**Background:**

Ceftolozane is a cephalosporin specifically developed to have enhanced antibacterial activity against *P. aeruginosa*. Combined with tazobactam, it was approved by FDA and EMA for hospital-acquired/ventilator-associated bacterial pneumonia. We evaluated trends in the activity of ceftolozane/tazobactam (C/T) against *P. aeruginosa* isolates collected as part of the global SMART surveillance program from patients with lower respiratory tract infections (RTI) in Argentina, Brazil, Chile, Colombia, and Mexico.

**Methods:**

In 2017-2020, 29 clinical labs each collected up to 100 consecutive gram-negative pathogens per year from patients with RTI. A total of 6,036 isolates were collected < 48 or ≥48 hours post-admission, of which 1,679 (27.8%) were *P. aeruginosa*. MICs were determined using CLSI broth microdilution and breakpoints. C/T- or imipenem-nonsusceptible isolates were screened for genes encoding β-lactamases.

**Results:**

Using only isolates from the 21 sites that participated in all 4 years, variability in susceptibility was seen over the study period among isolates collected < 48h post-admission, with a significant increasing trend in susceptibility only for C/T, while susceptibility was lower and more stable among isolates collected ≥48h (Figure). These patterns correlated with estimated carbapenemase rates, especially among isolates collected < 48h post-admission (9.7%, 4.2%, 8.1%, 1.6% carbapenemase-positive per year 2017-2020). Using isolates from all sites from the two most recent years to assess country-level susceptibility, substantial variability was seen. For example, susceptibility to C/T of isolates collected after 48h ranged from 62.5% for Chile (n=120) to ≥92% for Argentina (n=128) and Brazil (n=183), 12-31 percentage points higher than meropenem and piperacillin/tazobactam. This geographic pattern correlated with estimated carbapenemase rates (36% of isolates collected in Chile 48h post-admission versus ≤4% for Argentina and Brazil).

**Figure:**
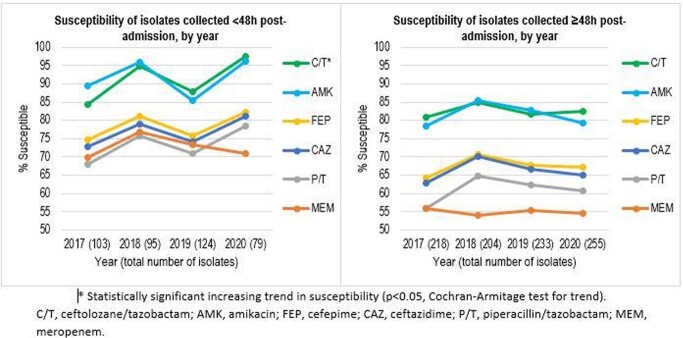

**Conclusion:**

Variability in antimicrobial susceptibility of *P. aeruginosa* from RTI was seen across Latin American countries, but overall no significant decreases in susceptibility were found over the study period. C/T remained the most active among the studied agents.

**Disclosures:**

**Fakhar Siddiqui, MD, MBA**, Merck & Co., Inc.: employee|Merck & Co., Inc.: Stocks/Bonds **Jacqueline Pavia, MD**, MSD COLOMBIA: EMPLOYEE **Charles A. DeRyke, PharmD**, Merck & Co., Inc. Merck Research Laboratories: Stocks/Bonds **Katherine Young, M.S.**, Merck & Co., Inc.: Stocks/Bonds.

